# *DNAH6*: A Newly Identified Gene Improving Total Number Born with Functional Enrichment Analysis in French Large White Pigs

**DOI:** 10.3390/ani16142158

**Published:** 2026-07-11

**Authors:** Lin Zhang, Junjie Shao, Chenchen Yang, Xiangdong Ding, Chenkai Liu, Chunmei Han, Weiping Ao

**Affiliations:** 1 College of Animal Science and Technology, Tarim University, Alar 843300, China; zhanglin10430@163.com (L.Z.); junjieshao191@163.com (J.S.); chen2yang0218@163.com (C.Y.); 2 College of Animal Science and Technology, China Agricultural University, Beijing 100193, China; xding@cau.edu.cn (X.D.); lck0808@163.com (C.L.); 3 Aksu Regional Agricultural Science and Technology Innovation Center, Aksu 843001, China

**Keywords:** French Large White sows, genome-wide association studies, total number born, *DNAH6*

## Abstract

Reproductive characteristics in pigs exhibit low heritability levels. Performing genome-wide association studies (GWAS) to pinpoint candidate genes for these characteristics is crucial for enhancing swine fertility. In this research, hair samples were gathered from 689 French Large White sows within the primary breeding population for DNA extraction purposes. Genotyping was carried out using a 50K liquid-phase SNP chip. Four statistical approaches—MLM, CMLM, FarmCPU, and BLINK—were utilized to perform GWAS on nine reproductive characteristics: total number born (TNB), litter birth weight (LBW), number born alive (NBA), number of healthy births (NHB), number of weak piglets (NWP), number of stillbirths (NSB), number of mummies (MUM), number of deformed fetuses (NDF), and adjusted 21-day litter weight (ALW). siRNAs targeting significantly associated genes were designed, and viral transfection was employed to introduce them into the ovarian granulosa cells of Large White sows to explore gene functions; transcriptome sequencing of granulosa cells treated with *DNAH6* siRNA was conducted to investigate the gene’s action mechanism. The analysis indicated that both MLM and CMLM models identified a significant locus linked to the total number of piglets born, with the SNP positioned at 3-60064515, associated with the *DNAH6* gene. Disruption of the *DNAH6* gene notably reduced the proliferation of ovarian granulosa cells (*p* < 0.05). Transcriptome sequencing results suggest that *DNAH6* may support the normal transcriptional activity of the cAMP signaling pathway by maintaining *EP300* and *CREBBP* expression in porcine ovarian granulosa cells, while potentially inhibiting the anti-proliferative effects of the FoxO pathway. This potential mechanism may help sustain the proliferative ability of granulosa cells and promote normal follicular development, which may ultimately contribute to an increase in the total number of piglets born in sows. These results offer a potential candidate marker for enhancing the total number born of French Large White sows.

## Simple Summary

The total number of piglets born is a crucial economic factor that affects the profitability of pig farming. Improving the reproductive efficiency of sows is vital for ensuring a consistent supply of pork. This research utilized French Large White pigs to investigate the impact and mechanisms of the *DNAH6* gene on the total number of piglets born. Findings revealed that the *DNAH6* gene promotes the growth of ovarian granulosa cells and supports normal follicle development, which in turn enhances the total number of piglets born to sows. This gene has the potential to act as a molecular marker for breeding sows with superior reproductive traits. The results offer a new target for molecular breeding in pigs, aiding in the enhancement of production efficiency and fostering the sustainable advancement of animal agriculture.

## 1. Introduction

The French Large White pig is a highly regarded breed for its maternal qualities, extensively utilized both in France and abroad. It is recognized for its remarkable features, including exceptional reproductive capabilities, strong maternal instincts, rapid growth, efficient feed conversion, and adaptability to various environments. A key reproductive characteristic in pigs is the total number of piglets born (TNB), and enhancing this trait can lead to a significant rise in the availability of market-ready hogs, thus ensuring a consistent supply of premium pork to satisfy increasing consumer demand. Nonetheless, the reproductive traits of sows tend to have low heritability and are significantly affected by environmental conditions, rendering conventional phenotypic selection methods less effective for genetic enhancement [1]. Consequently, investigating the genetic foundations of these reproductive traits is essential for refining breeding strategies, boosting sow reproductive efficiency, and increasing the industry’s economic returns.

Conventional methods for breeding traits with low heritability are often lengthy, and the results are greatly influenced by environmental factors, which complicates the process of achieving precise genetic enhancements. Genome-wide association studies (GWAS) facilitate the analysis of complex traits by examining genome-wide single nucleotide polymorphisms (SNPs), allowing for the accurate identification of significant loci and genotypes associated with reproductive traits in pigs at the molecular DNA level, thus enhancing selection precision [2,3]. In 2011, Onteru [4] et al. utilized the Illumina PorcineSNP60 BeadChip to study lifetime reproductive characteristics in Large White pigs and crossbred sows, pinpointing the *SLC22A18* gene on *SSC2* as a primary candidate for TNB and number born alive (NBA). Following this, Hidalgo [5] et al. applied GWAS to explore the genetic foundations of the gestation period in pigs, identifying *HBEGF* as a key gene influencing gestation length within the quantitative trait locus (QTL) region on chromosome 2 in Dutch Landrace pigs, which is vital for embryo implantation and maintaining pregnancy. A meta-analysis by Sell-Kubiak [6] et al. across five pig populations revealed an association between the *PRKD1* gene and both number of stillborn piglets (NSB) and TNB. Conversely, the *SOSTDC1* gene, linked to TNB, has been shown to negatively impact male fertility in various mammalian species. Research from China has indicated that the *LPAR3* gene is significant in regulating early pregnancy and enhancing embryo implantation rates and is a crucial candidate gene for the high reproductive capacity of local breeds like Erhualian pigs [7]. Zhang [8] et al. performed a comprehensive analysis of healthy and weaned litters in multiparous Large White sows, identifying candidate genes such as *BLVRA*, *STK17A*, *PSMA2*, and *C7orf25*, which are involved in essential biological functions like sperm motility, spermatogenesis, and meiosis. The following year, Hong Yf [9] et al. identified nine reproductive traits in Large White sows through ssGWAS, ultimately recognizing *GPR12* on *SSC11* as a significant candidate gene related to TNB. The variations in quantitative trait loci (QTLs) and candidate genes affecting reproduction are notable across different pig breeds. These genes are involved in critical reproductive processes and biological pathways, including ovarian follicle development, selection of dominant follicles, ovulation, and embryo implantation, by modulating the production and release of hormones related to reproduction [10,11]. The efficiency of embryo implantation and the status of ovarian development together influence the potential TNB of sows [12,13]. Nonetheless, there is a gap in multi-model GWAS joint analyses regarding the genetic mechanisms of reproductive traits in purebred French Large White pigs, necessitating further systematic research to confirm candidate genes and clarify their regulatory functions.

The 50K liquid SNP chip, created by Professor Ding Xiangdong’s research group at China Agricultural University, enhances the identification of crucial loci across 8800 known economic trait QTL regions, utilizing specific probes for genome-wide screening. It exhibits exceptional sensitivity in detecting loci with minor effects, offering dependable molecular markers and effective breeding approaches for swine genetics [14]. This study aims to use the aforementioned 50K liquid chip to genotype 689 French Large White sows with complete phenotypic records of reproductive traits. This study aims to identify key genetic loci and candidate genes associated with reproductive traits in French Large White pigs and further explore the biological functions and potential mechanisms of core genes. The findings are expected to provide novel genetic markers and theoretical support for molecular breeding of high reproductive performance in this breed.

## 2. Materials and Methods

### 2.1. Experimental Animals and Reproductive Performance

The experimental population was obtained from a breeding pig farm in Aksu, Xinjiang. All experimental animals were raised under standardized management, with uniform dietary nutrition levels and feeding environmental conditions. A total of 689 healthy French Large White pigs aged 20–24 months were selected as the research subjects. Nine reproductive traits were recorded: total number born (TNB), litter birth weight (LBW), number born alive (NBA), number of healthy births (NHB), number of weak piglets (NWP), number of stillbirths (NSB), number of mummies (MUM), number of deformed fetuses (NDF), and adjusted 21-day litter weight (ALW). This study used SPSS 27.0 software for quality control of phenotypic data, removing duplicates, missing values, and outliers. Outliers were eliminated using the mean ± 3 times standard deviation method, and corrected phenotypic values were calculated [15]. To eliminate the interference of environmental and grouping factors, we further calculated least squares means (LS-means) via R 4.2.1. The mixed model included fixed effects of parity, farm and farrowing year-season so as to obtain unbiased phenotypic values for follow-up analysis.

### 2.2. Sample Collection

#### 2.2.1. Genomic DNA Extraction Sample

Hair tissue samples were collected from 689 experimental sows. Genomic DNA of French Large White pigs was extracted using a DNA extraction kit (TIANGEN, Beijing, China), with detailed procedures following the manufacturer’s instructions. After extraction, the integrity of the DNA was assessed by 1.5% agarose gel electrophoresis, while DNA purity and concentration were measured using a NanoDrop 2000 spectrophotometer (Thermo Fisher Scientific, Waltham, MA, USA). Qualified DNA samples were then sent to Beijing Compass Biotechnology Co., Ltd. (Beijing, China) for genotyping using the GBTS 50K liquid chip developed by China Agricultural University (Beijing, China).

#### 2.2.2. Granulosa Cell Samples

Fresh ovaries were collected from 6 healthy French Large White pigs (aged 36–48 months), immediately placed in a sterile thermos cup containing 37 °C physiological saline (with 100 IU/mL penicillin and 50 mg/mL streptomycin), and transported to the laboratory within 3 h. The ovaries were rinsed three times with 75% alcohol and physiological saline respectively. Follicular fluid containing granulosa cells (GCs) was aspirated from 3 mm-diameter follicles using a 1 mL sterile syringe, and GCs were collected.

### 2.3. Test Methods

#### 2.3.1. Quality Control

The raw sequencing data in FASTQ format were aligned to the pig reference genome, *Sus scrofa* 11.1 (https://www.ensembl.org/Sus_scrofa/Info/Index, Sscrofa11.1; accessed on 6 June 2025) using BWA-MEM2 v2.2.1 software [15,16]. The alignment results were sorted and deduplicated using SAMtools v1.9, and SNP detection was performed through the HaplotypeCaller module of GATK v4.1.4 [17]. Stringent data quality control was performed using PLINK1.9 software [18]. SNPs with individual call rates > 95%, SNP call rates > 90%, minor allele frequencies > 5%, and without significant deviation from Hardy–Weinberg equilibrium (*p* > 1 × 10^−6^) were retained. SNPs that were not correctly mapped to the reference genome or located on sex chromosomes were removed, and samples with close kinship were excluded [19]. The high-quality SNPs retained after these quality control procedures were subsequently used for genome-wide association analysis.

#### 2.3.2. Genome-Wide Association Analysis of Reproductive Traits

Genome-wide association analysis was performed using four statistical models (MLM, CMLM, FarmCPU, and BLINK) implemented in the GAPIT3 package of R version 4.2.1. For the nine reproductive traits of French Large White sows, we conducted independent GWAS procedures separately for each trait and applied a uniform genome-wide significance threshold across all traits to ensure consistent statistical standards among different trait analyses so as to screen for SNP loci significantly associated with sow reproductive traits. The Bonferroni correction method was used to determine the genome-wide significance threshold: after quality control, 52,000 high-quality autosomal SNPs were retained, and *p* < 9.6 × 10^−7^ was taken as the genome-wide significance threshold. The CMplot v4.2.0 package was used to generate Manhattan plots and QQ plots to visualize the distribution of *p*-values. The BEDTools v2.30.0 program was utilized to screen for genes located within 500 kb upstream and downstream of the significant SNP loci. Candidate genes were annotated using the NCBI database (https://www.ncbi.nlm.nih.gov/; accessed on 19 July 2025).

#### 2.3.3. Culture and Identification of French Large White Pig GCs

GCs were resuspended in DMEM complete medium containing 10% fetal bovine serum, inoculated and cultured at 37 °C with 5% CO_2_ for 48 h. The cells were fixed with 4% paraformaldehyde, permeabilized with 0.2% Triton X-100 (Biotopped, Beijing, China), and blocked with 5% BSA (Sigma-Aldrich, St. Louis, MO, USA). Rabbit anti-FSHR antibody (1:200, Abcam, Cambridge, UK) was added and incubated at 4 °C overnight. After PBS washing, Alexa Fluor 488-labeled secondary antibody (1:500, abcam, Cambridge, UK) was added and incubated at room temperature for 1 h. Nuclei were counterstained with DAPI (Biotopped, Beijing, China) before mounting and observed under a fluorescence microscope. PBS was used instead of the primary antibody as a negative control.

#### 2.3.4. *DNAH6* siRNA Transfection in Granulosa Cells

Three pairs of independent siRNAs targeting different exonic regions of the DNAH6 gene (siRNA1, siRNA2, siRNA3) with non-overlapping sequences and one pair of negative control (NC) siRNAs were designed and synthesized by Shanghai Hanbio Biotechnology Co., Ltd. (Shanghai, China). The siRNA sequences are listed in Table 1. The in vitro transfection of GCs was performed according to the instructions of Lipofectamine 2000 transfection reagent (Invitrogen, Carlsbad, CA, USA) [20]. All transfection assays and subsequent functional experiments were conducted with three independent biological replicates; each biological replicate was set with three technical replicates to ensure the reliability and reproducibility of the results. The interference efficiency of *DNAH6* siRNA was detected by qPCR. The specific qPCR primers for *DNAH6* and the reference gene GAPDH are listed in Table 2.

#### 2.3.5. Effect of DNAH6 on GC Proliferation

The GCs from the experimental group with the best interference efficiency, the negative control group (si-NC), and the untransfected control group (Control) were subjected to a cell viability assay using the Cell Counting Kit-8 (CCK-8), with three biological replicates set for each group. At 20, 36, 48, 60, 72, 84, and 96 h of culture, 10 μL of CCK-8 reagent was added to each well, followed by incubation for 1 h before measuring the optical density (OD value) at a 450 nm wavelength using a microplate reader. The cell growth curve was plotted with culture time as the *x*-axis and OD value as the *y*-axis.

#### 2.3.6. Transcriptome Sequencing of GCs After DNAH6 siRNA Interference

Ovarian granulosa cells with optimal interference efficiency and blank control samples were selected, with three biological replicates set for each group, and sent to Wuhan BGI for transcriptome sequencing. The raw sequencing data underwent base calling using CASAVA v1.8.2 software and were converted to FASTQ format. SOAPnuke v1.5.6 software was employed to filter the raw reads, removing those containing adapters and low-quality reads (with >20% of bases having quality value Q5% N bases [21]). The high-quality clean reads obtained after filtering were aligned to the pig reference genome (*Sus scrofa* 11.1) using HISAT2 v2.2.1 software [22]. Quality control metrics included Q30 (proportion of bases with quality score ≥ 30), mapping rate, and correlation between biological replicates. Differential expression gene analysis was performed using DESeq2 v1.38.3 software, with screening criteria set at |log2FC| ≥ 1 and FDR < 0.05 [23]. The clusterProfiler v4.6.2 software was employed for Gene Ontology functional enrichment analysis and KEGG pathway enrichment analysis of differentially expressed genes [24,25].

#### 2.3.7. Statistical Analysis

Statistical analysis of the data was performed using SPSS 27.0 software, and graphs were generated using GraphPad Prism 10 software. The relative expression levels in qPCR results were calculated using the 2^−ΔΔCt^ method [26]. Data are presented as mean ± standard deviation, and one-way ANOVA was used for significance analysis. Significant differences were denoted as * (*p* < 0.05), and highly significant differences were denoted as ** (*p* < 0.01).

## 3. Results

### 3.1. Reproductive Performance of French Large White Sows

Raw data of nine reproductive traits from 689 French Large White sows were processed and quality-controlled. Phenotypic records covered parity 1 to parity 4, with 2093 to 2413 valid observations per trait. Descriptive statistics based on corrected least squares means ± SE are presented in Table 3. Detailed results of the normality test are provided in Appendix A.1.

### 3.2. Sequencing Quality Analysis and Quality Control

**Figure 1:**
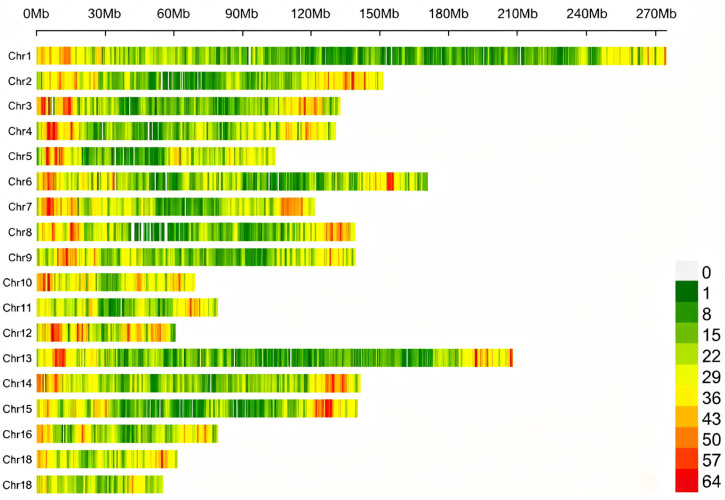
SNP density distribution map.

Genotyping for single nucleotide polymorphisms (SNPs) across the entire genome was conducted on 689 individuals from the primary population of French Large White pigs. The alignment of sequencing reads to the reference genome achieved an impressive rate of over 99.4% for every individual. Following rigorous quality control measures, 52,000 high-quality SNP loci were selected for further analysis. Figure 1 illustrates the distribution of these SNP loci across the chromosomes, revealing a consistent distribution pattern without notable segmental deletions or density imbalances. These findings suggest that the quality of the genotyping data is robust and appropriate for future genome-wide association studies (GWAS).

### 3.3. Genome-Wide Association Study of Reproductive Traits

Genome-wide association analysis (GWAS) was performed on nine reproductive traits of French Large White sows, including total number born (TNB), litter birth weight (LBW), number born alive (NBA), number of healthy piglets (NHB), number of weak piglets (NWP), number of stillbirths (NSB), number of mummified fetuses (MUM), number of deformed fetuses (NDF), and adjusted 21-day litter weight (ALW). Four statistical models (MLM, CMLM, FarmCPU, and BLINK) were employed for the association analysis. We calculated the genomic inflation factor (λGC) for all trait-model combinations; most λGC values fell within the reasonable quality control range of 0.93 to 1.01, and only a few combinations showed mild *p*-value inflation or contraction. The overall quality control results met the requirements of genome-wide association analysis. Detailed λGC values for each combination are provided in Table S2 in the Supplementary Materials. The genome-wide significance threshold was determined using the Bonferroni correction method (α = 0.05), with an adjusted threshold of 9.6 × 10^−7^ calculated based on the 52,000 quality-controlled autosomal SNPs retained in this study. A total of 17 SNP loci that meet the Bonferroni-corrected genome-wide significance criterion were identified, distributed across eight chromosomes (chromosomes 1, 2, 6, 12, 14, 15, 16, and 17). Among these loci, 5 were repeatedly detected by at least two statistical models, which substantially improved the reliability of the association results.

**Figure 2:**
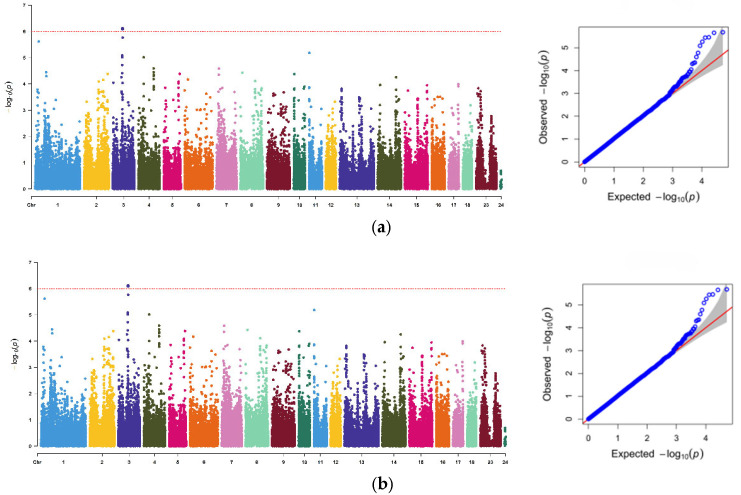
GWAS results for the TNB trait in French Large White pigs: (**a**) MLM model, (**b**) CMLM model.

**Figure 3:**
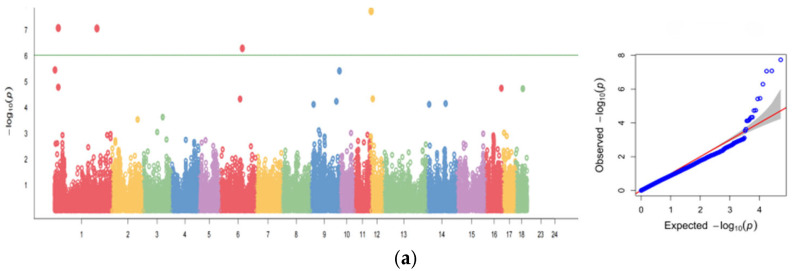

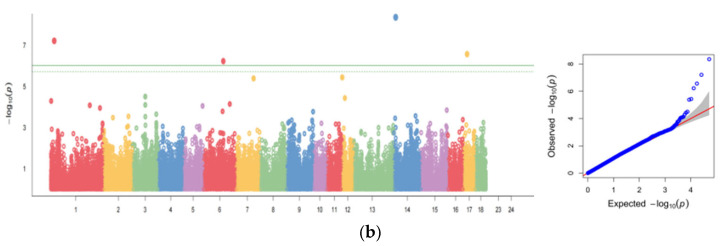
GWAS results for the LBW trait in French Large White pigs: (**a**) FarmCPU model, (**b**) BLINK model.

**Figure 4:**
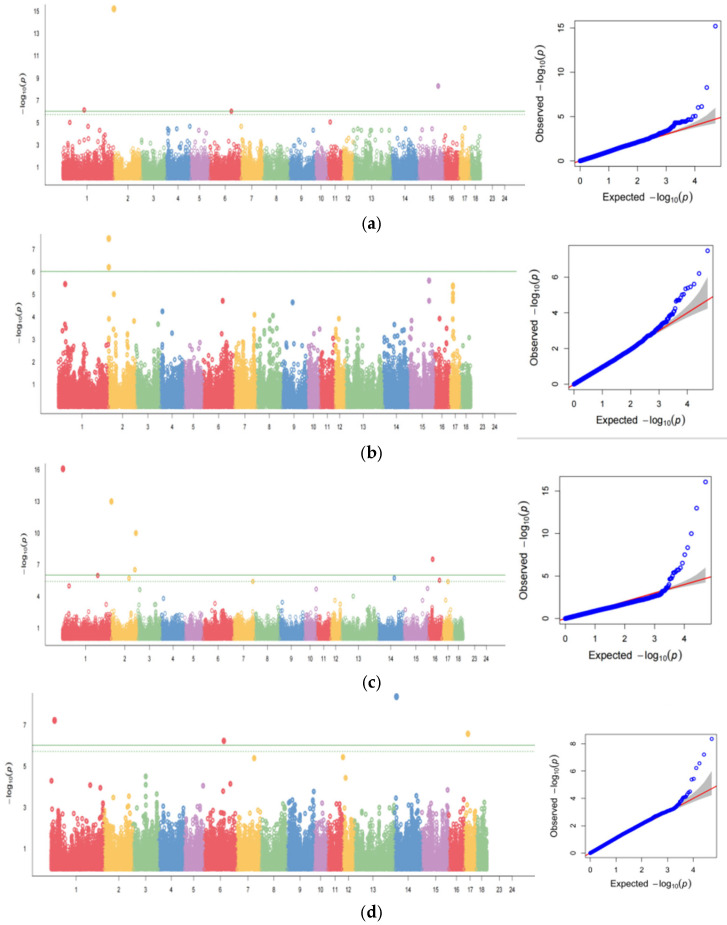
GWAS results for NDP in French Large White pigs: (**a**) BLINK model, (**b**) CMLM model, (**c**) FarmCPU model, (**d**) MLM model.

Notably, the MLM and CMLM models identified a significant locus associated with TNB on chromosome 3 (SNP position: 3:60064515), with the candidate gene *DNAH6*. Meanwhile, the FarmCPU and BLINK models detected significant loci associated with LBW, including the locus 14:112206429 on chromosome 14 (candidate gene *TLX1*) and 1:18614836 on chromosome 1 (candidate gene *ADGB*). In addition, *MYL12A*, *CETN1*, and *CCDC40* were also identified as candidate genes significantly associated with LBW. On chromosome 2, four SNP loci associated with weak piglet number were identified, with candidate genes including *TBXT*, *GDF9*, *GFRA3*, *PLCXD3*, *MNS1*, *CDKN1C*, and *PHLDA2* (detailed in Table 4 and Figure 2, Figure 3 and Figure 4, and Appendix A.2).

### 3.4. Effect of DNAH6 siRNA Interference on GC Proliferation

#### 3.4.1. Identification of In Vitro Cultured Primary GCs from French Large White Pigs

**Figure 5:**
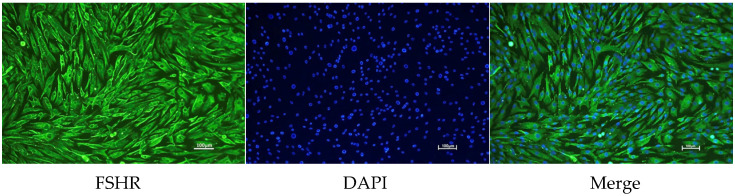
Immunofluorescence identification of primary granulosa cells from French Large White pigs.

The results from the FSHR immunofluorescence assay indicated that the nuclei of GCs, stained with DAPI, displayed a blue fluorescence. In addition, distinct green fluorescence was detected in the cytoplasmic region, which confirmed the effective expression of the FSHR protein. This also validated that the primary cells that were isolated and cultured were, in fact, ovarian granulosa cells, as illustrated in Figure 5.

#### 3.4.2. Interference Effect of DNAH6 siRNA

**Figure 6:**
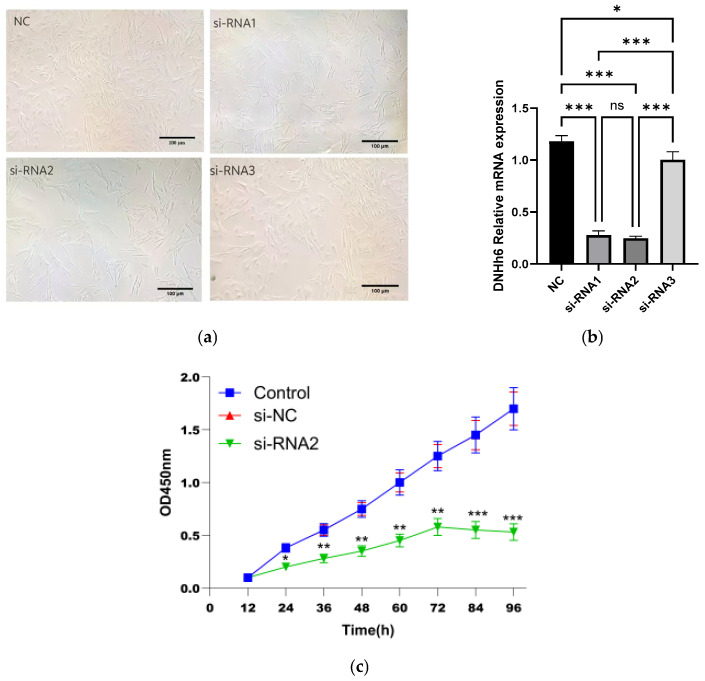
Effects of the *DNAH6* gene interference on ovarian granulosa cells in French Large White pigs. (**a**) Growth status of GCs before and after *DNAH6* siRNA interference; (**b**) expression changes in *DNAH6* after siRNA interference; (**c**) cell growth at different stages after CCK-8 interference. *, **, and *** indicate *p* < 0.05, *p* < 0.01, and *p* < 0.001, respectively; ns indicates no significant difference.

Twenty-four hours post-transfection with *DNAH6* siRNA, observations made using an inverted microscope indicated that the three transfection groups had a reduced number of adherent cells and a lower cell density compared to the control group, with si-RNA2 showing the most pronounced effect. Refer to Figure 6a. The impact of the three *DNAH6*-targeting si-RNAs (si-RNA1, si-RNA2, and si-RNA3) was assessed through qPCR analysis. The findings revealed that all three si-RNAs significantly decreased the expression of the *DNAH6* gene in comparison to the negative control group (*p* < 0.05), the si-RNA2 group exhibited the best interference efficiency of 81.4% (*p* < 0.01). Consequently, si-RNA2 was chosen for further experimental investigations. See Figure 6b.

The growth of GCs was assessed using the CCK-8 assay from 12 to 96 h following *DNAH6* interference. The proliferation patterns for both the blank control and negative control (si-NC) groups were nearly identical, demonstrating a steady increase in cell growth throughout the culture period. Conversely, the si-RNA2 interference group exhibited a marked reduction in GC proliferation relative to the control groups, with this suppression becoming more pronounced as time progressed, as illustrated in Figure 6c.

### 3.5. Transcriptome Analysis of the DNAH6 Gene in Ovarian Granulosa Cells Interfered by si-RNA2

**Figure 7:**
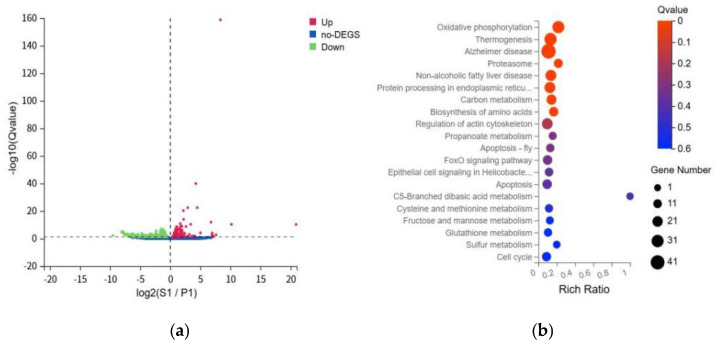
Results of differential gene identification and enrichment analysis. (**a**) Volcano plot of differentially expressed genes after *DNAH6* interference in ovarian granulosa cells; (**b**) bubble chart of KEGG enrichment analysis.

**Figure 8:**
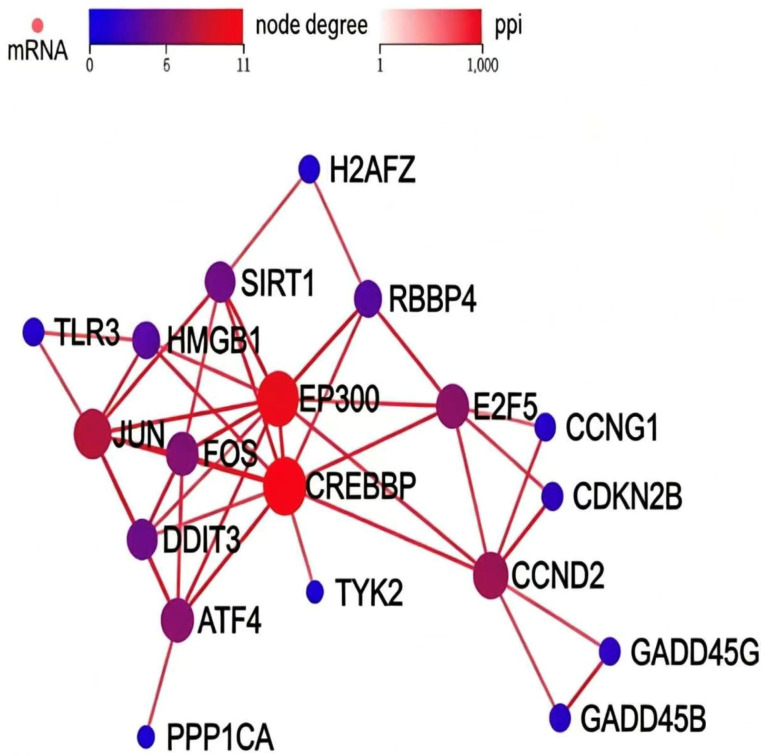
PPI network diagram.

Analysis of transcriptome sequencing in ovarian granulosa cells following *DNAH6* interference identified 1001 genes with altered expression levels. Among these, 545 genes were found to be upregulated, including notable examples like *CDKN2B*, *TLR3*, *SELENO*, and *RPL10A*, while 456 genes were downregulated, featuring *EP300*, *CREBBP*, *CAPN7*, and *ATP5J2*. KEGG pathway analysis indicated significant enrichment of these differentially expressed genes in both the cAMP and FoxO signaling pathways, as illustrated in Figure 7. Furthermore, the protein–protein interaction (PPI) network analysis indicated that the differentially expressed proteins in granulosa cells created an interaction network primarily focused on *EP300* and CREBBP, which closely interacted with transcription factors such as *JUN*, *FOS*, *ATF4*, and *SIRT1*, along with cell cycle regulators like *CCND2*, *CDKN2B*, and members of the *GADD45* family, as depicted in Figure 8.

## 4. Discussion

In this study, we performed genome-wide association analysis on swine reproductive traits using four statistical models and identified a total of 17 significant SNP loci, which were mainly distributed across 8 chromosomes: chromosomes 1, 2, 6, 12, 14, 15, 16 and 17. A single statistical model is susceptible to interference from factors such as population stratification and environmental effects, which may lead to biased association results [27]. Therefore, we adopted a multi-model joint analysis strategy to improve the robustness of the results. It should be noted that the core reason for the discrepancy in detection results among different models lies in the differences in their statistical frameworks. MLM and CMLM are classical single-locus mixed linear models that test only one SNP at a time, with high detection sensitivity for loci with moderate genetic effects. In contrast, FarmCPU and BLINK belong to multi-locus iterative models, which iteratively incorporate background significant SNPs as fixed effects into the model to control genome-wide false positives. However, this design dilutes the signal intensity of moderate-effect loci and reduces detection sensitivity for such loci. In this study, 5 loci were validated by at least two models, and the cross-model consistency significantly improved the reliability of the association results. For example, the DNAH6 locus associated with the total number born reached the Bonferroni-corrected genome-wide significance threshold in both MLM and CMLM models but was not detected by FarmCPU and BLINK—this is precisely because its moderate genetic effect was diluted by the multi-locus iterative framework. The significant loci associated with the number of deformed fetuses were predominantly concentrated on chromosome 2, suggesting that this region may harbor key genomic segments regulating abnormal fetal development in swine. For the three traits of total number born, litter birth weight and number of deformed fetuses, we identified key candidate genes respectively: *DNAH6* for total number born; *TLX1*, *ADGB* and *CCDC40* for litter birth weight; and *CDKN1C*, *PHLDA2*, *TBXT*, *GDF9*, etc. for number of deformed fetuses.

The recently identified gene DNAH6, linked to the total number of offspring, is a significant component of the dynein protein family. It is essential for various physiological functions, including the control of ciliary and flagellar movements, as well as the development of left-right asymmetry in embryos. Multi-omics data from the PigGTEx v2.0 public database show that *DNAH6* is moderately expressed in porcine ovarian tissue and specifically highly expressed in ovarian granulosa cells. Ovarian cis-eQTL analysis revealed that intragenic SNPs of *DNAH6* can significantly modulate gene expression. The SNP 3-60064515 identified in this study is such a cis-regulatory variant, which may affect porcine reproductive traits by altering gene expression. However, the cis-eQTL effect of this lead SNP has not been validated in our own Large White breeding herd; that requires further verification with matched genotype and expression data in follow-up work.

Current research on the *DNAH6* gene mainly targets diseases related to human cilia and male infertility, with no studies addressing its potential impact on reproductive characteristics in pigs. Li [28] et al. found that dysfunctional *DNAH6* can lead to visceral heterotaxy by impairing ciliary movement, presenting symptoms akin to primary ciliary dyskinesia (PCD). This research was pioneering in connecting *DNAH6* to defects in organ laterality and disorders of ciliary motility. Following this, several investigations have uncovered harmful mutations in *DNAH6* among male infertility patients exhibiting multiple morphological abnormalities of the sperm flagella (MMAF), establishing that defects in *DNAH6* can result in irregularities in sperm tail structure and motility issues [29,30]. Additionally, Shao [31] et al. identified new pathogenic variants in *DNAH6*, noting acrosomal anomalies and irregular chromatin condensation in sperm from individuals with these variants. Patients with MMAF experience a loss of normal motility and fertilization ability due to malformations in sperm flagella; however, they can successfully achieve fertilization through intracytoplasmic sperm injection (ICSI), which enhances conception and pregnancy rates. Overall, these findings indicate that *DNAH6* plays a role in reproductive functions by influencing the structure and performance of cilia and flagella [32]. Nevertheless, its involvement in female reproductive health, especially regarding ovarian granulosa cell activity and litter size, is still not well understood.

Following the introduction of *DNAH6* siRNA into porcine ovarian granulosa cells cultured in vitro, transcriptome sequencing analysis indicated a significant enrichment of differentially expressed genes within the cAMP and FoxO signaling pathways. Additionally, protein–protein interaction (PPI) network analysis revealed that the differentially expressed proteins created an interaction network focused on EP300 and CREBBP. Under typical physiological conditions, the cAMP signaling pathway plays a crucial role in regulating the proliferation and differentiation of granulosa cells (GCs), as well as steroid hormone production and follicular growth [33]. When follicle-stimulating hormone (FSH) attaches to the surface receptors on GCs, it triggers adenylate cyclase activation, resulting in an increase in intracellular cAMP levels, which in turn activates protein kinase A (PKA). PKA facilitates the phosphorylation of the transcription factor CREB and also phosphorylates the FoxO transcription factor. The phosphorylated *CREB* then recruits its coactivators, *EP300* and *CREBBP*, which act as essential transcriptional coactivators downstream of the cAMP pathway, enhancing the expression of reproduction-related genes through their histone acetyltransferase (HAT) activity, thus contributing to the maintenance of GCs’ functional balance [34]. Conversely, phosphorylated FoxO is confined to the cytoplasm, losing its ability to activate transcription [35,36]. The FoxO signaling pathway mainly governs cell cycle progression and proliferation. When activated, it can increase the expression of *CDKN2B* while inhibiting *CCND2*, leading to G1-phase cell cycle arrest in GCs and reducing their proliferation capacity [37,38]. This disruption hinders normal follicular development and maturation, lowers ovulation rates, and ultimately results in fewer piglets being born to sows.

This study only infers the involvement of *DNAH6* in the regulation of the cAMP signaling pathway based on transcriptomic differential expression data and has not yet conducted functional validation experiments such as cAMP content, protein phosphorylation, and luciferase reporter assays. The regulatory mechanism still needs to be further confirmed by subsequent experiments.

## 5. Conclusions

Through multi-model genome-wide association analyses, this study identified a significant association between the *DNAH6* gene and the total number born in French Large White pigs. In porcine ovarian granulosa cells, *DNAH6* may sustain the expression of *EP300* and *CREBBP* to maintain normal transcriptional output of the cAMP signaling pathway and concurrently suppress the anti-proliferative activity of the FoxO pathway. This potential regulatory pattern may preserve the proliferative capacity of granulosa cells, support normal follicular development, and ultimately contribute to an improved total number born in sows. This study provides a novel potential candidate gene for the genetic improvement of litter size traits toward high fertility in French Large White pigs.

## Figures and Tables

**Table 1:** siRNA primer sequences.

Primer Name	Primer Sequence
*DNAH6*-siRNA1	F: GGAAGAAGUUAGUGCUGAATT
	R: UUCAGCACUAACUUCUUCCTT
*DNAH6*-siRNA2	F: AGUUCUAGAUGAUCUUCAATT
	R: UUGAAGAUCAUCUAGAACUTT
*DNAH6*-siRNA3	F: GGACUUUGUUGUAUUGAAATT
	R: UUUCAAUACAACAAAGUCCTT
NC	F: UUCUCCGAACGUGUCACGUTT
	R: ACGUGACACGUUCGGAGAATT

**Table 2:** Primer information.

Primer	Sequence	Annealing Temperature/°C	Fragment Length/bp	Accession Number
*DNAH6*	F: CTGCCTGGACAGATGAACCC R: TAAGAGAAAAGCGACACTTGGC	60	373	XM_021087305.1
*GAPDH*	F: TGTTTGTGATGGGCGTGAACAA R: ATGGCGTGGAGCAGTGGTCATAA	58	154	NM_001034034.2

**Table 3:** Statistics of sow reproductive traits.

Trait	TNB (Piglets)	NBA (Piglets)	NSB (Piglets)	MUM (Piglets)	NDF (Piglets)	NHB (Piglets)	NWP (Piglets)	LBW (kg)	ALW (kg)
Sample Size	2283	2325	2200	2413	2093	2378	2384	2133	2343
Least Squares Means ± SE	16.22 ± 2.16	13.89 ± 2.57	0.83 ± 1.48	0.39 ± 0.87	0.03 ± 0.28	12.38 ± 4.64	1.31 ± 1.60	18.91 ± 3.12	80.00 ± 11.56

TNB, total number born; NBA, number born alive; NSB, number of stillborn; MUM, number of mummies; NDF, number of deformed fetuses; NHB, number of healthy births; NWP, number of weak piglets; LBW, litter birth weight; ALW, adjusted 21-day litter weight.

**Table 4:** SNP loci associated with reproductive traits in French Large White pigs.

Gene	SNP	Chr	Pos	*p*-Value	Traits	Model
*DNAH6*	3_60064515	3	60064515	2.03 × 10^−7^	TNB	MLM, CMLM
*TLX1*	14_112206429	14	112206429	1.53 × 10^−7^	LBW	BLINK, FarmCPU
*ADGB*	1_18614836	1	18614836	6.18 × 10^−8^	LBW	BLINK, FarmCPU
*MYL12A*	6_103409730	6	103409730	5.98 × 10^−7^	LBW	BLINK
*CETN1*	6_105350960	6	105350960	5.13 × 10^−7^	LBW	FarmCPU
*CCDC40*	12_2269903	12	2269903	1.85 × 10^−8^	LBW	FarmCPU
*TBXT*	1_2916708	1	2916708	8.57 × 10^−17^	NDP	FarmCPU
*GDF9*	2_134779503	2	134779503	3.01 × 10^−7^	NDP	FarmCPU
*GFRA3*	2_140461869	2	140461869	1.02 × 10^−10^	NDP	FarmCPU
*PLCXD3*	16_26205018	16	26205018	3.09 × 10^−8^	NDP	FarmCPU
*MNS1*	1_115357083	1	115357083	7.38 × 10^−7^	NDP	BLINK
*CDKN1C*	2_2055699	2	2055699	6.26 × 10^−7^	NDP	FarmCPU, MLM, CMLM, BLINK
*PHLDA2*	2_2135799	2	2135799	6.19 × 10^−16^	NDP	FarmCPU, CMLM, MLM

## Data Availability

The datasets presented in this study are not publicly available because the related project is not yet completed and the data will be used in follow-up research. Requests to access the datasets should be directed to the corresponding author.

## Supplementary Materials

The following supporting information can be downloaded at https://www.mdpi.com/article/10.3390/ani16142158/s1, Table S1: The KEGG and GO enrichment results of differentially expressed genes; Table S2: The genomic inflation factor (λGC).

## Appendix A

### Appendix A.1

Normal distribution test of 9 reproductive traits data in French Large White pigs.

### Appendix A.2

Genome-wide association analysis.

**Traits**	**Total Number Born**	**Number Born Alive**	**Number of Healthy Births**	**Number of Weak Piglets**	**Number of Stillborn**	**Number of Deformed Fetuses**	**Number of Mummies**	**Litter Birth Weight**	**Adjusted 21-Day Litter Weight**
grapheme	AAA	BBB	CCC	DDD	EEE	FFF	GGG	HHH	III

MLM model.

CMLM model.

FarmCPU model.

Blink model.
